# Digitalisation of information and management optimisation in Multiple Victim Incidents. Analytical study

**DOI:** 10.1371/journal.pone.0303247

**Published:** 2024-05-14

**Authors:** Navid Behzadi Koochnai, Raúl Muñoz Romo, Nicolás Riera López, Rafael Caballero Cubedo, Soledad Gómez de la Oliva, Teresa Martin de Rosales Cabrera, Almudena Castaño Reguillo

**Affiliations:** 1 Servicio de Urgencias Médicas de la Comunidad de Madrid (SUMMA112), Madrid, Spain; 2 Fundación para la Investigación e Innovación Biosanitarias en Atención Primaria (FIIBAP), Madrid, Spain; 3 Facultad de Ciencias de la Salud, Universidad Camilo José Cela de Madrid, Madrid, Spain; Universitair Kinderziekenhuis Koningin Fabiola: Hopital Universitaire des Enfants Reine Fabiola, BELGIUM

## Abstract

**Introduction:**

Triage is a crucial tool for managing a Multiple Victim Incident (MVI). One particularly problematic issue is the communication of results to the chain of command and control. Favourable data exists to suggest that digital triage can improve some features of analogue triage. Within this context we have witnessed the emergence of the Valkyries Project, which is working to develop strategies to respond to MVIs, and especially cross-border incidents. To that end, an IT platform called “SIGRUN” has been created which distributes, in real time, all the information to optimise MVI management. A full-scale simulation, held on the Spain-Portugal border and featuring contributions from different institutions on both sides of the border, put to the test the role of information digitalisation in this type of incidents.

**Objective:**

To evaluate the impact of the synchronous digitalisation of information on the optimal management of Multiple Victim Incidents.

**Method:**

Clinical evaluation study carried out on a cross-border simulation between Spain and Portugal. A Minimum Data Set (MDS) was established by means of a modified Delphi by a group of experts. The digital platform “SIGRUN” integrated all the information, relaying it in real time to the chain of command and control. Each country assigned two teams that would carry out digital and analogue triage synchronously. Analogue triage variables were gathered by observers accompanying the first responders. Digital triage times were recorded automatically. Each case was evaluated and classified simultaneously by the two participating teams, to carry out a reliability study in a real time scenario.

**Results:**

The total duration of the managing of the incident in the A group of countries involved compared to the B group was 72.5 minutes as opposed to 73 minutes. The total digital assistance triage (AT) time was 37.5 seconds in the digital group, as opposed to 32 minutes in the analogue group. Total evacuation (ET) time was 28 minutes in the digital group compared with 65 minutes in the analogue group. The average differences in total times between the analogue and the digital system, both for primary and secondary evaluation, were statistically significant: p = 0.048 and p = 0.000 respectively. For the “red” category, AT obtained a sensitivity of 100%, also for ET, while with regard to AT safety it obtained a PPV of 61.54% and an NPV of 100%, and for ET it obtained a PPV of 83.33% and an NPV of 100%. For the analogue group, for AT it obtained a sensitivity of 62.50%, for ET, 70%, for AT safety it obtained a PPV of 45.45% and an NPV of 92.31%, while for ET it obtained a PPV of 70% and an NPV of 92.50%. The gap analysis obtained a Kappa index of 0.7674.

**Conclusion:**

The triage system using the developed digital tool demonstrated its validity compared to the analogue tool, as a result of which its use is recommended.

## Introduction

In recent decades, the increase in Multiple Victim Incidents (MVI) and disasters where the health and emergency services have been overwhelmed, at least initially, by the number of victims they had to treat [1] highlights the need for transversal strategies such as those stated in the Sendai Framework for Disaster Risk Reduction 2015–2030 [2].

The optimisation of management of this type of incidents translates into an increase in survival. One of the most commonly-used tools for improving MVI management and saving a greater number of lives is that of triaging [3]. This optimisation of management involves classification, prioritisation and distribution of resources bearing in mind not only the severity of the patients’ situation but also the prognosis for their injuries, survival, quality of life and the resources available to achieve the best results for all of the victims [4–6].

There are many types of triage, but they all share the same principle of victim prioritisation and management. One of the most-commonly used triage models is START (Simple Triage and Rapid Treatment), which is based on a system that divides patients up into four categories: patients classified as “red” present life-threatening injuries requiring immediate treatment, patients classified as “yellow” present serious injuries but their treatment can wait, “green” patients present minor injuries, and “black” patients are those that have either passed away or are expected to do so [7].

However, several studies have questioned the correct execution of a triage during an MVI [8,9]. Errors in the triage process such as under-triage (which may result in patients not receiving the care they need within the proper time) and over-triage (which means the patient is classified with a higher severity level) represent an improper use of resources and in both cases results in an increase in mortality [4].

Another very common problem in the triage process is the communication of results to the entire care chain and the chain of command and control of the MVI in order to be able to properly manage the necessary resources [10,11]. Analogue recording of data and the need to communicate same in a non-synchronous manner, whether it is by telephone or TETRA (Terrestrial Trunked Radio), produces a cyclical and inopportune arrival of information [12,13].

Some studies have offered encouraging data regarding digital triage which can, at least partly, improve certain crucial aspects of analogue triage, such as: reducing the triage execution time with greater sensitivity and specificity [14], reducing treatment time for more severe patients thanks to less managing time and more effective medical decisions [15], better accessibility and universality of healthcare in communities with limited resources, such as rural areas [16] and facilitating medical research thanks to its practicality in terms of data gathering, thus helping to create algorithms and protocols more efficiently, among other points [17].

However, we need to continue to make progress in the research until we can integrate these systems into our standard practice [18]. In a recent meta-analysis, Wallace WH, et al. also state that we need to carry on researching this area given the variability of results and low accuracy of studies [19].

Within this context, the strategic lines of transnational research of the Horizon Europe-H2020 projects have resulted in the emergence of the Valkyries project (grant agreement no. 101020676), which aims to develop, integrate and demonstrate capabilities that can enable an immediate, co-ordinated response to emergencies, including search and rescue, health and safety, in natural/man-made disaster scenarios with multiple victims, and with particular application to cross-border incidents.

To achieve the aforementioned objectives, an IT platform by the name of “SIGRUN” has been created that integrates and relays to the care chain and the chain of command and control, in real time, all the basic essential information, known as the Minimum Data Set (MDS), in order to optimise managing of the MVI. The MDS was developed by an expert committee using the modified Delphi method.

A full-scale simulation, held on the Spain-Portugal border in May 2023, with more than 50 victims and featuring the participation of over 400 servicepeople and members of different organisations responsible for managing emergencies put to the test the role of information digitalisation in this type of incident. The simulation involved the participation of the emergency departments of each of the two countries.

### Main objective

To evaluate the impact of the synchronous digitalisation of information on the optimal management of Multiple Victim Incidents.

### Specific objectives

- To evaluate and validate the digital triage system as compared with analogue triage.
- To evaluate the impact of the synchronous digitalisation of information on reducing the time of analogue communication by the chain of command and control of the Multiple Victim Incident.

## Method

A clinical evaluation study carried out in a large scale, cross-border simulation between Spain and Portugal and with the direct involvement of both countries’ emergency departments and with more than 400 servicepeople and members of different organisations on the ground.

The following points were considered during the preparatory stage:

- Creating the Chain of Command and Control: a group of experts established the chain of command and control with the heads of the Triage Post—Advanced Health Post (AHP)–Evacuation Post–Health Command Post (HCP)–Incident Management Team (IMT) of the coordinating centre.

Establishing the Minimum Data Set (MDS): using the modified Delphi method, an expert committee defined the MDS to be used for the optimal management of the MVI. The items we selected to that end were: the feasibility of collecting the data, real-time onward communication of the data to the whole chain of command and control, and being considered essential to good management of an MCI. Subsequently, the process was completed using the Utstein method to improve data quality, which was modified appropriately for formal systematic collection of essential information in a public health emergency. The process had three phases. In the first one, the essential principles for governing the whole process were selected: being considered essential for the proper management of MCIs in cross-border areas, adequate capacity to collect and report the data without any need to change the protocols and procedures of first responders, the proficiency and experience of experts (at least 20 years’ experience in the field of knowledge), ensuring the implementation of the operational procedures of first responders and taking into account the maximum degree of agreement between experts. In the second phase, the items were voted on in line with the literature. In the third phase, the Delphi method was established through three rounds of voting by the group of experts, eliminating the items that did not obtain any votes. To ensure that a consensus was obtained, the degree of agreement between the experts was determined by calculating Cohen’s Kappa.

- Defining the incident and victims: an expert committee agreed on such items as type of incident, number of victims, pathologies of same, colour code for care triage and evacuation and their clinical evolution, among other points.

**Fig 1:**
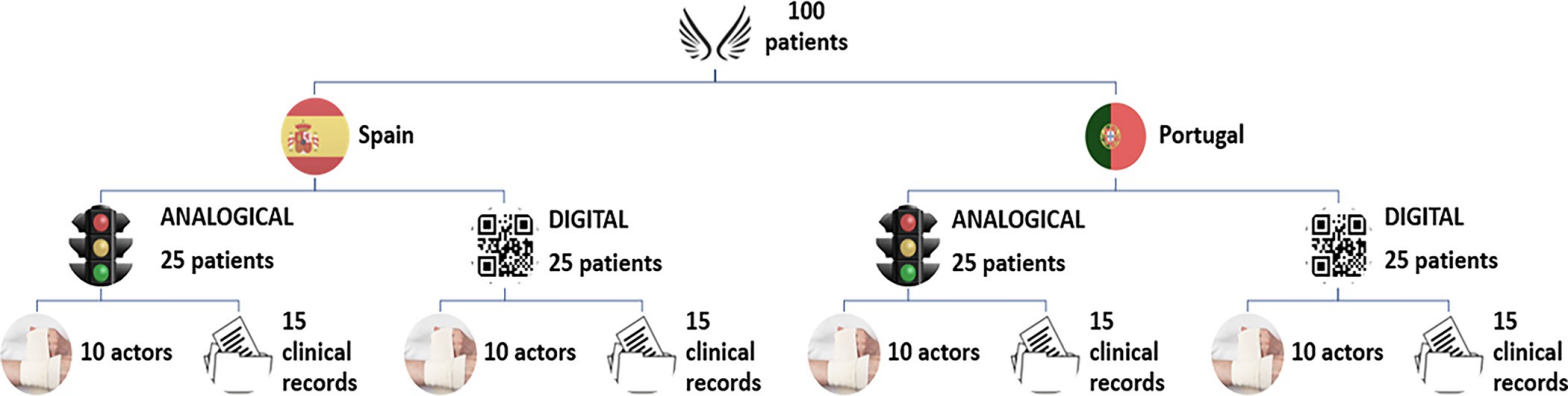
Study case/patient distribution algorithm.

**Fig 2:**
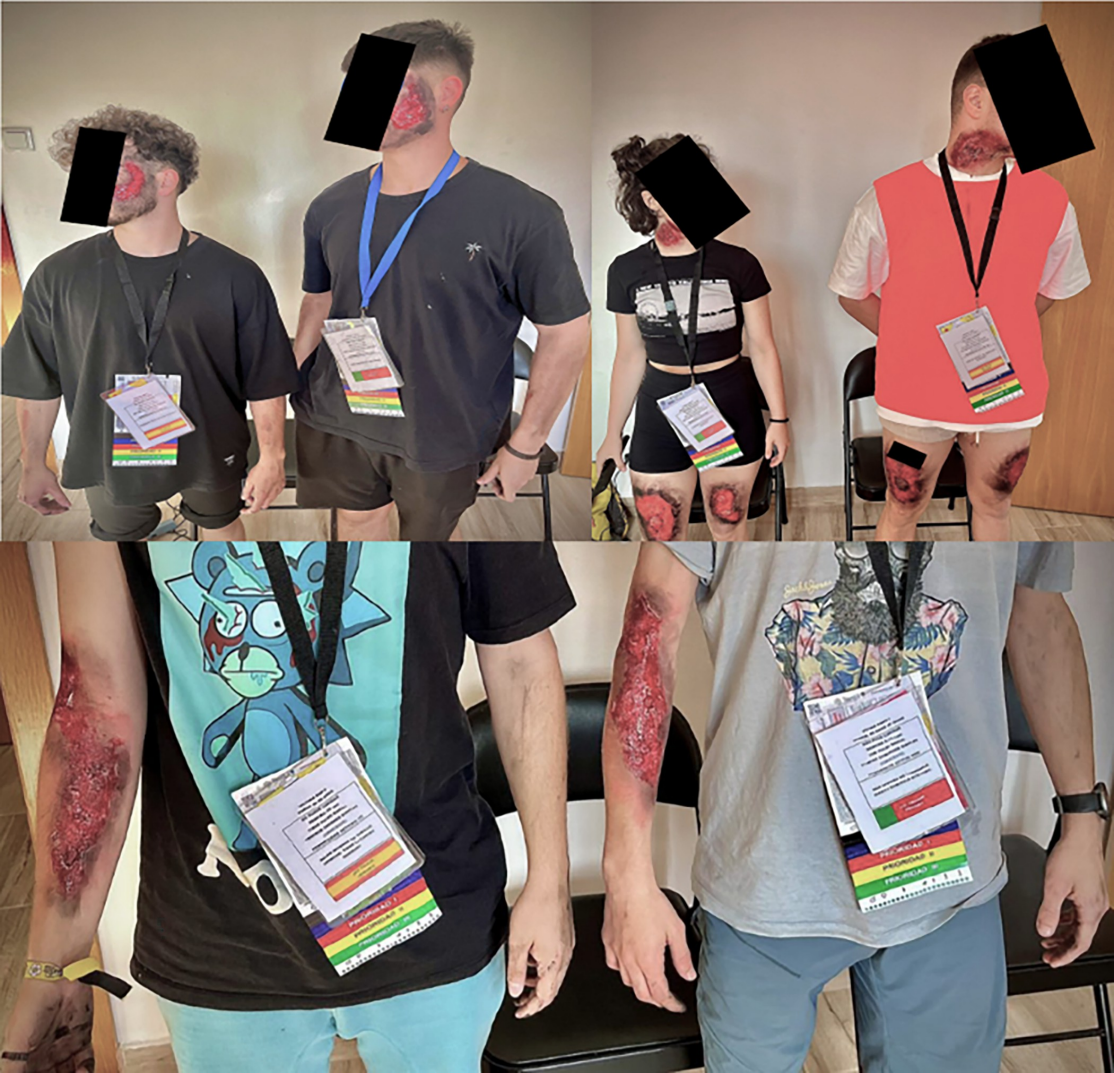
Examples of characterisation of volunteers.

Each country assigned two teams to carry out, in parallel and without interfering with each other, digital and analogue triage for 25 assigned victims. The victims (identical on both sides of the border) were represented on the day of the simulation by 10 actors, while 15 clinical records provided clinical information and two triage files, one analogue (from the country that treated the patient) and the other with a QR code to digitalise the information gathered, with analogue notes of same being made in the event of a digital malfunction (which did not occur). See Figs 1 and 2.

- Digital platform for the synchronous gathering and distribution of information: a modular, multilayer, high-security digital platform called SIGRUN, developed specifically for this project, integrated all the information from the MDS and relayed it in real time to the entire chain of command and control.
- System for gathering variables: more than 10 observers of the project accompanied the first responders and the command heads in order to monitor the analogue communications and triage times. The digital triage time was recorded automatically.

With regard to analysis of the data, this study was organised into two clearly differentiated stages:

### Validating the digital triage system as compared with the analogue triage system

Usually, when designing a study aimed at calculating or sometimes estimating the basic indicators to evaluate the effectiveness of a diagnostic test, an N-sized sample is taken of a specific population to which the screening test has been applied, and the criteria of truth for making the estimates. In the present study, this sample was determined by the volunteers who took part in the drill where the management of the cross-border incident was staged. To that end, a sample was selected of N1 sick patients and another of N2 non-sick individuals, diagnosed as such by the benchmark test, applied to N individuals (N1 + N2), thus creating a 2x2 table, and in this way evaluating the diagnostic test.

Analysis and validation of the strategy was carried out by adhering to the minimum requirements for the publication of diagnostic tests (STARD initiative) [20]. Thus, basic indexes were calculated: sensitivity (S), specificity (SP) and positive and negative predictive values (PPV and NPV). In the same way, other indicators were calculated such as the validity index (VI) or correct hit ratio, and the Youden index (YI) [21], or the ratio for the rectified probability of detecting illness.

To calculate these values, we needed to select a “Gold Standard” that would define patients’ real severity levels, given that without this definition, according to Windle et al [22], it is not possible to test and validate a triage system. In our study, we establish as a benchmark the traditional analogue system executed by experts, as we stated previously (Table 1).

**Table 1:** Expert committee’s Gold Standard for selecting the colour for each stage.

PATIENT	TRIAGE R	TRIAGE Y	FINDINGS
1	R	R	2^nd^ DEGREE BURNS ON THE FACE, NASAL MUCOSA AND OROPHARYNX
2	R	R	2^nd^ DEGREE BURNS ON THE NECK, ABDOMEN AND BOTH LEGS (> 25% TBSA)
3	R	R	COLLAPSE OF RIGHT HEMITHORAX ON INHALATION, DEFORMITY OF LEFT THIGH
4	R	R	ANISOCORIA (RIGHT MYDRIASIS), CONTUSION INJURY IN THE FRONTAL AREA
5	Y	Y	TACHYPNEIA. ABRASIONS ON THE HANDS
6	Y	R	SIGNS OF PELVIC TRAUMA, WITH UNSTABLE PELVIS
7	Y	Y	LEG DEFORMED TO PAINFUL DEGREE
8	Y	Y	2^nd^ DEGREE BURNS ON THE THORAX AND ARMS (AROUND 18% TBSA) FUNCTIONAL INCAPACITY OF RIGHT ANKLE
9	Y	Y	DEFORMITY WITH FUNCTIONAL INCAPACITY OF SECOND THIRD OF RIGHT THIGH
10	Y	Y	INJURY ON PERONEAL MALLEOLUS WITH SIGNIFICANT DEFORMITY AND SWELLING
11	G	G	SUPERFICIAL CONTUSION INJURY ON RIGHT FRONTAL AREA
12	G	G	CONTUSION AND EROSIONS ON BOTH KNEES
13	G	G	EROSIONS ON HEAD AND FACE, SOME WITH SMALL HAEMORRHAGES
14	G	G	CONTUSION INJURY OF APPROXIMATELY 4 CM ON LEFT EYEBROW
15	G	Y	EROSION AND BRUISING ON THE CHEST WALL
16	G	G	EROSIONS ON FACE AND THORAX
17	G	G	EROSION OF LEFT ARM
18	G	G	LACERATION OF APPROXIMATELY 10 CM ON RIGHT ARM
19	G	G	VARIOUS ABRASIONS
20	G	G	INJURY ON SCALP (4 CM) AND ON RIGHT ARM (5 CM), ABRASIONS ON LEG
21	B		
22	B		
23	B		
24	B		

*Note*: B: Black; R: Red; Y: Yellow; G: Green.

S and SP are the traditional basic measurements of the diagnostic value of a test. They measure the diagnostic discrimination of a test in relation to a benchmark criterion which is considered the truth. These indicators allow us in principle to directly compare the effectiveness of a test with that of others, and to expect similar results when they are applied in different countries, regions, or settings. S indicates the ability of the test to detect a sick subject; that is, it expresses how "sensitive" the test is to the presence of the illness or condition. To quantify its expression, probabilistic terms are used. SP indicates the ability of the test to identify as healthy (not sick) those who are. The PPV would be the probability that a truly urgent subject had been classified as urgent (red colour), and the NPV would be the probability that a non-urgent subject had been classified as non-urgent (yellow and green colours).

With regard to managing the exercise, it was established that each case should be evaluated and classified simultaneously by the two participating teams, so as to be able to determine the reliability of the tests. This enabled us to carry out a reliability study in real time and scenario, and not in hypothetical situations. Using this premise, we also proceeded to apply the evaluation of a test for the two triage tools via the parallel method [23], whereby all these tests are applied simultaneously to the same sample of individuals, so that all individuals that obtain negative results in all tests are considered to be negative, and all others positive.

Finally, as a complement to validity, we examined the consistency between the benchmark and the valuations of the assigned teams. In this way, we obtained the corresponding Cohen’s Kappa indexes [24] for the evaluation of the consistency level.

To solve the problem due to a part of the observed agreement (in principle unknown) that may be attributable exclusively to chance, we use the kappa index developed by Cohen in 1960. Its purpose is to quantify the degree of agreement once the part that can be attributed is eliminated, exclusively at random. This index relates the agreement that observers exhibit, beyond that which is due to chance, with the potential agreement also beyond chance. To do this, we calculate the difference between the proportion of observed agreement and the proportion of agreement expected by chance. If this is equal to zero, then the degree of agreement that has been observed can be attributed entirely to chance. If the difference is positive, this indicates that the degree of agreement is greater than what would be expected if only chance were operating and vice versa: in the (admittedly unlikely) case in which the difference was negative then the data would be exhibiting less agreement than that which is expected only by chance. Kappa is the quotient between that quantity and the maximum agreement that can be expected without the intervention of chance. This index, when the observers are independent, takes the value 0, and reaches the maximum value of 1 only if there is perfect agreement between the observers and finally, it is never less than –1. To identify what kappa value can be considered as an indicator of good agreement, in 1972 Landis and Koch proposed a scale for interpreting the kappa value that considers a value greater than or equal to 0.40 as acceptable and values greater than 0 as excellent 0.75 [25].

Evaluation of the triage tools was carried out at several different levels: first of all, the ability to classify a patient correctly as “red”, both in the primary and the secondary evaluation, and secondly the ability to classify a patient correctly as “yellow”, both in the primary and the secondary evaluation. It was suggested that for the *urgent levels* (red and yellow), there should be no discrepancies, given that these were the most severe patients. Thus, it was decided that an analysis should be carried out by separating both levels and leaving *non-urgent levels* (green and black) grouped together. Likewise, to assess the safety of the triage system, we focused on predictive values, both PPV and NPV. These severity levels were validated given that they were considered critical for responding to the triaging objectives.

### Evaluating the impact of the synchronous digitalisation of information on reducing analogue communication time for the chain of command and control of the Multiple Victim Incident

To do this, we set up a clinical evaluation study with a total sample size of 100 clinical records. Once the simulation had been carried out, we evaluated the total time spent managing the incident and the congestion of telecommunications systems bearing in mind two independent groups with quantitative variables applying the Mann-Whitney U test (Wilcoxon signed-rank test of related samples) [26]. This test enabled us to determine whether there were any differences in the averages between the groups we were comparing, owing to the different ways the data was distributed, which as they involved time variables, their behaviour was different to normal. Furthermore, due to the nature of the study and that the sample and its size were determined by the logistical conditions of the exercise, the statistical power of the study was calculated. The power of a hypothesis test is the probability that the test correctly rejects the null hypothesis. Therefore, in some studies with negative results it will be concluded that there are no differences when there really are. This error is known as a type II error. The probability of making an error of this type is usually denoted by β and its complement, 1-β, which is what is known as statistical power or statistical power [27].

Subsequently, we attempted to monitor the possible confusion effect produced by those factors which, as they are related to the exposure factor in the study, determine the appearance of the result through stratification via the participant groups (countries A and B), an effect that had already been observed previously (consistency between observers).

### Ethical and legal aspects of the project

The participating volunteers who simulated patients were students of the Degree in Health Emergency Technician in Extremadura, Spain. In the simulation exercise, the participating actors were recruited by the General Directorate of Emergencies of Extremadura (Spain), who informed and obtained their verbal consent to participate in the exercise, not by the Valkyries project. The head of the Valkyries project is aware of this consent from the General Directorate of Emergencies of Extremadura, in compliance with ethical and legal requirements. None of them received any financial reward for their activities. We individually collect their consent to participate to the Use-Case providing the information on the project and on the dissemination activities. The consent was freely given by all participants and nobody revoked it. All the activities followed the internal procedures of the simulation activities, which are fully compliant with the applicable legal framework. As only simulated data were used and no personal or patient data were required, the study did not need to be assessed by an Ethics Committee beforehand. The VALKYRIES Project did not deal with personal data. The Consortium of VALKYRIES was made up of ethical-legal experts, who were involved in both compliance, regulatory and standardization tasks, and a declaration was signed by those responsible for the Consortium, showing the commitment to comply with all ethical-legal issues. Within this declaration, the decision of the Consortium to develop the work without hiring volunteers, not processing personal data, is stated. The case study scenarios involved first responders to receive feedback on the effectiveness of the interoperable platform through the application of simulated and unreal data. The simulated data were generated directly by the Consortium.

## Results

### Of the evaluation and validation of the triage system

The corresponding data for S and SP were obtained, which are shown in Table 1, with the digital Care Triage system VALKYRIA (VALK CT) obtaining an S for the “red” category of 100%, and for the “yellow” category of 41.67%. For the Digital Evacuation Triage VALKYRIA (VALK ET) obtained an S for the “red” category of 100%, and for the “yellow” category of 58.33%. With regard to the safety of the care Triage System, for the groups of cases identified as “red” and “yellow”, we estimated a PPV of 61.54% and an NPV of 100%, compared with an estimated PPV of 71.43% and an NPV of 72%, respectively, which means that Non-Urgent cases were identified, a priori, by the Triage System as Non-Emerging, with an 18% margin of error, and 0% for the Emerging cases. With regard to ET, for the groups of cases identified as “red” and “yellow”, a PPV of 83.33% was estimated, compared to an estimated PPV of 87.50% and an NPV of 77.27%, respectively, which means that the Non-Urgent cases were identified, a priori, by the triage system as Non-Emerging, with a 13% margin of error and 0% for the Emerging cases for the digital system **(**VALK ET).

With regard to the Analogue Care Triage tool (ANALOG CT), we obtained an S for the “red” category of 62.50%, and 83.33% for the “yellow” category, in the CT. For the Analogue Evacuation Triage (ANALOG ET) we obtained an S for the "red" category of 70%, and 58.33% for the “yellow” category. As for safety in the ANALOG CT system, for the groups of cases identified as “red” and “yellow”, a PPV of 45.45% and an NPV of 92.31% were estimated for the former, and a PPV of 47.62% and an NPV of 81.82% for the latter, respectively, which means that Non-Emerging cases were identified as such, a priori, by the triage system, with an 18.18% margin of error, and 52.38% for Emerging cases. For the ANALOG ET, for the groups of cases identified as “red” and “yellow”, a PPV of 70% and an NPV of 92.50% were estimated for the former, and a PPV of 87.50% and an NPV of 77.27% for the latter, respectively, which means that the Non-Emerging cases were identified as such, a priori, by the triage system, with a 30% margin of error, with 7.50% for Emerging cases and 22.73% for cases that can wait and 12.50% for delayed Urgencies.

The results of the parallel testing showed, for VALK ET and ANALOG ET, an S for “red” levels of 100% in Evacuation Triage, with an estimated PPV of 66.67% and an NPV of 100%, respectively, which means that Non-Emerging cases were identified as such, a priori, by the triage system, with a margin of error of 0%, and of 33.33% for the Emerging cases if we assume that both tests confirm the benchmark result being applied at the same time by the two teams. For the “yellow” levels, an S of 75% was obtained, with an estimated PPV of 90% and an NPV of 85% respectively, which means that the Non-Urgent cases were identified as such, a priori, by the triage system, with a 10% margin of error and 15% for the Emerging cases under the same assumption. The rest of the results are shown in Table 1. As we can see, for the CT, these values show results that are quite significantly poorer when both tools are applied at the same time by the two teams.

To complement the criterion validity, an examination was made between the benchmark and the valuations of the different triage systems. Table 2 shows the results of the gap analysis. For these data, a Kappa Index was obtained of 0.7674 and 0.6591, respectively, for the detection of “red” and “yellow” cases, a good consistency according to the criteria of Landis-Koch [25], which means that the triage systems facilitated a good triaging level assignation for the classified patients.

**Table 2:** Comparative analogical and digital triage times between the groups of the two countries.

Healthcare Triage Median in Seconds	COUNTRY A	COUNTRY B	
Analogue Method	34.5		31
Digital Method	34.5		49
Evacuation Triage Median in Seconds			
Analogue Method	62.5		77
Digital Method	23.5		32
Total triage time in minutes			
Healthcare Triage	38		31
Evacuation Triage	61		41
Average patient transfer times in minutes			
Time Interval Healthcare Triage-Evacuation Triage	50.1		28
Time interval Evacuation Triage-Evacuation to Hospital	13.5		32
Time interval Healthcare Triage-Hospital Arrival	72.5		73
Total incident	193		210
Telephone or TETRA communication times in minutes			
Healthcare Triage-Command and Control station	4.2		18.4
Evacuation Triage-Command and Control station	38.5		27
Total Analogue Communication	42.7		45.5

### Of the evaluation of the results of the exercise

The total duration of managing the incident for the group of participating countries A compared to group B was 193 minutes as opposed to 210 minutes.

The total time for Care Triage was 38 minutes for country A group compared to 31 minutes for country B. A difference was found between the primary triage times separated by colour-coded severity levels, tending towards a reduction of the average time spent. In the VALK group (“red”: 32 seconds, “yellow”: 49 seconds, “green”: 41 seconds) compared to the ANALOG group (“red”: 45.5 seconds, “yellow”: 34.5 seconds, “green”: 30 seconds).

The total time for secondary triage was 28 minutes in the VALK group compared to 65 minutes in the ANALOG group. The secondary triage times separated by colour-coding were significantly different between the VALK group (“red”: 41.5 seconds, “yellow”: 33 seconds, “green”: 28 seconds) and the ANALOG group (“red”: 115 seconds, “yellow”: 170 seconds “green”: 60 seconds).

Congestion times in communications for the benchmark system were analysed separately in the Triage Post and in the Advanced Health Post for groups A and B.

The total congestion time in communications in the Triage Post was 252 seconds for the primary evaluation and with a line-engaged period of 2310 seconds in the Advanced Command Post for the secondary evaluation for group A. Meanwhile, for group B, a period of 1108 seconds was observed for the primary evaluation, and with a line-engaged period of 1625 for the secondary evaluation.

The total line-engaged time for communications in the Advanced Health Post with the Coordinating Centre was 42.7 minutes for group A and 45.5 minutes for group B. All of the stratified results for the participating groups are shown in Table 3.

**Table 3:** Comparative correspondence in analogue triage vs digital by means of labels.

Patient reference	Healthcare Triage					Evacuation Triage				
	Reference	*COUNTRY A*		*COUNTRY B*		Reference	*COUNTRY A*		*COUNTRY B*	
		Analogue Method	Digital Method	Analogue Method	Digital Method		Analogue Method	Digital Method	Analogue Method	Digital Method
1	**R**	R	R	R	R	**R**	R	R	Y*	R
2	**R**	R	R	Y*	R	**R**	R	R	R	R
3	**R**	R	R	R	R	**R**	R	R	R	R
4	**R**	Y*	R	Y*	R	**R**	R	R	Y*	R
5	**Y**	R*	G*	Y	Y	**Y**	Y	G*	Y	Y
6	**Y**	Y	R*	Y	R*	**R**	Y*	R	R	G*
7	**Y**	G*	R*	Y	Y	**Y**	R*	R*	G*	G*
8	**Y**	Y	Y	Y	G*	**Y**	R*	Y	Y	Y
9	**Y**	Y	R*	Y	Y	**Y**	Y	Y	G*	Y
10	**Y**	Y	R*	Y	Y	**Y**	R*	R*	Y	Y
11	**G**	G	G	Y*	G	**G**	G	G	G	G
12	**G**	Y*	G	Y*	G	**G**	G	G	G	G
13	**G**	G	G	Y*	G	**G**	G	G	G	G
14	**G**	G	G	Y*	G	**G**	G	G	G	G
15	**G**	Y*	Y*	Y*	G	**Y**	Y	Y	Y	Y
16	**G**	G	G	G	G	**G**	G	G	G	G
17	**G**	G	G	Y*	G	**G**	G	G	G	G
18	**G**	Y*	Y*	Y*	G	**G**	Y*	Y*	G	G
19	**G**	G	G	G	G	**G**	G	G	G	G
20	**G**	G	G	Y*	G	**G**	G	G	G	G
21	**B**	B	B	R*	B	**B**	B	B	B	B
22	**B**	B	B	R*	B	**B**	B	B	B	B
23	**B**	B	B	R*	B	**B**	B	B	B	B
24	**B**	B	B	R*	B	**B**	B	B	B	B
25	**B**	B	B	R*	B	**B**	B	B	B	B

*Note*: B: Black; R: Red; Y: Yellow; G: Green;

*: Error respect triage reference.

In the evaluation of the statistical significance of the differences between the averages of total times found by applying the Wilcoxon signed-rank test for related samples, both for the primary and the secondary evaluation, a difference in average times was obtained between the ANALOG and the VALK systems with a statistical significance of p = 0.048 for the primary evaluation and p = 0.000 for the secondary evaluation. Therefore, we can conclude that the differences found are statistically significant.

Regarding this point, it must be taken into account that non-parametric tests usually offer less statistical power than their corresponding parametric significance tests, so with non-parametric tests the probability of rejecting the null hypothesis is lower while the alternative hypothesis is true. For all that, we thought it would be interesting to calculate the power of the study. Generally, one tends to work with a power of around 80% or 90%. Thus, we finally obtain with our data: If we want to detect a minimum difference of 7 seconds: Bilateral approach: 81.29% as static power. If we want to detect a minimum difference of 5 seconds: Bilateral approach: 52.98% as static power. Therefore, we observe that to detect a difference of about 7 seconds in the management of the incident, the power of the test is quite adequate, as we have commented, but for minor differences it decreases considerably. Due to the nature of the study, we believe that these differences of less than 7 seconds are not very relevant. Therefore, we can affirm that the power of the test can be considered quite adequate to respond to the purposes of the study [27].

Finally, we evaluated once again the differences found by stratifying for the participant groups, given that this factor, as we noted previously, can act as a possible confusion factor. Thus, we obtained an average difference of times between the ANALOG and the VALK systems with a statistical significance, for group A, of p = 0.548 for the primary evaluation and p = 0.000 for the secondary evaluation; for group B we obtained an average difference of times between the ANALOG and the VALK systems with a statistical significance of p = 0.038 for the primary evaluation and p = 0.000 for the secondary evaluation. Therefore, we can conclude that for group A, during the primary evaluation, no differences in statistical significance were found. The difference between the raw value of the Wilcoxon signed-rank test for related samples and the value obtained for each group confirmed the confusion effect that the variable exerts, as a result of which it was stratified. Table 4 shows the percentage of correct assignment of suit colour to the most critical Red and Yellow patients.

**Table 4:** Percentage of correct assignment of suit colour to the most critical red and yellow patients.

Parameters	Healthcare Triage				Evacuation Triage			
	*Red Triage*		*Yellow Triage*		*Red Triage*		*Yellow Triage*	
	Digital Method	Analogue Method	Digital Method	Analogue Method	Digital Method	Analogue Method	Digital Method	Analogue Method
**Sensitivity (%)**	100	62.5	41.67	83.33	100	70	58.33	58.33
**Specificity (%)**	88.1	85.71	90	45	95	92.5	94.44	94.44
**Positive Predictive Value (%)**	61.54	45.45	71.43	47.62	83.33	70	87.5	87.5
**Negative Predictive Value (%)**	100	92.3	72	81.82	100	92.5	77.27	77.27

## Discussion

In various studies, the validity of triage has been assessed based on the predictive capacity for hospitalisation, patient death and length of stay, in relation to the level assigned. In our study, owing to the nature of the type of triage and scope, we opted to assess criterion validity through the concept of S and SP, understanding that triaging functions as a screening tool for classifying patients. Moll stresses the importance of a triage system having a high sensitivity to identify those patients whose condition might worsen if they do not receive correct treatment [28–30].

As a first step, we observe that the digital triage tool differentiates between Emerging patients (who cannot wait), and Non-Emerging patients (who can wait), which is why it is important to know the values of Sensitivity (100%), Specificity (88.10%) and Negative Predictive Value (100%); in this case all of these are above 80%, which are considered to be good values, both in Care Triage and in Evacuation. This was not the case for Urgency as such, which showed lower S values in both evaluations.

The fact that the S of the test and the NPV are very high corroborates the idea that the model designed is safe, given that it minimises the phenomenon of infra-triage, a situation that is necessary in a triage model in order that the most severe patients should be seen as soon as possible, thus minimising the probability that their treatment is delayed.

The PPV shown for both evaluations signifies that a significant proportion of patients classified as emerging (red) and urgent (yellow), were not in fact so. In principle this does not affect these patients negatively, but strategies must be sought to improve these values, given that a greater number of patients mistakenly classified as emerging and urgent can affect the amount of treatment time given to less urgent cases (green).

In the same way, the NPV was analysed for each of the levels, with slightly low values detected for cases classified as “yellow” level in both evaluations, with hardly any differences observed with the analogue tool. For the “red” level, the NPV was always 100% for the digital tool, but not for the analogue one which, even though it delivered good figures, never reached 100% in either of the evaluations.

Furthermore, the parallel evaluation tests showed that in the event that both tools agreed, they did not especially enhance the effectiveness of the triage, which would support the observation of the non-equivalence in value of the two tools.

The gap analysis carried out was used for the comparison between observers, without actually assessing at that moment which of the two were carrying out under-triage or over-triage, with the aim of evaluating the reliability of the system. In this way we obtained “very good” data according to Landis-Koch criteria for both groups to compare in the detection of Emerging and acceptable cases in the case of Urgent cases for Evacuation Triage. We should point out that the different composition of both teams in terms of training levels could be one of the determining factors for the poor results obtained for the Evacuation Triage.

Another fundamental point to bear in mind is the impact of the synchronous digitalisation of information on the managing of MVI by the chain of command and control. In the analogue case, the heads of the chain of command and control of the MVI had to speak on the telephone or via TETRA for an average of 44 minutes to obtain the crucial information, which represents a significant delay in management given that the information is not being transmitted in real time. However, in the digital case, the information was synchronous and shared between the entire chain of command and control, including the receiving hospitals, who were able to prepare themselves well in advance for the type and number of patients that would be arriving within one or two hours. Another important point is the maximum traceability of patients and their respective data from minute 0, which helps to improve the management of each case.

## Conclusions

After having evaluated the Triage System through the developed digital tool, its validity has been demonstrated compared to the analogue tool, as a result of which its use is recommended. The Triage System using said tool shows good Sensitivity and Specificity for Emerging patients (“red” category) and Urgent patients (“yellow” category), though Sensitivity is significantly lower for the latter. The high Negative Predictive Value makes it highly effective as a screening tool for emerging cases. Patients classified as Emerging in care triage and evacuation triage showed the best results, as a result of which we can state that the Triage ensures special attention for these patients.

The synchronous digitalisation of information reduces analogue communication to the minimum, thereby maximising situational awareness of the incident among the chain of command and control, which results in the optimal management of the incident as a whole and possible improvement in terms of victim survival.
